# Characterization of the MCM homohexamer from the thermoacidophilic euryarchaeon *Picrophilus torridus*

**DOI:** 10.1038/srep09057

**Published:** 2015-03-12

**Authors:** Kasturi Goswami, Jasmine Arora, Swati Saha

**Affiliations:** 1 Department of Microbiology, University of Delhi South Campus, Benito Juarez Road, New Delhi 110021, India

## Abstract

The typical archaeal MCM exhibits helicase activity independently *in* *vitro*. This study characterizes MCM from the euryarchaeon *Picrophilus torridus*. While PtMCM hydrolyzes ATP in DNA-independent manner, it displays very poor ability to unwind DNA independently, and then too only under acidic conditions. The protein exists stably in complex with PtGINS in whole cell lysates, interacting directly with PtGINS under neutral and acidic conditions. GINS strongly activates MCM helicase activity, but only at low pH. In consonance with this, PtGINS activates PtMCM-mediated ATP hydrolysis only at low pH, with the amount of ATP hydrolyzed during the helicase reaction increasing more than fifty-fold in the presence of GINS. While the stimulation of MCM-mediated helicase activity by GINS has been reported in MCMs from *P.furiosus, T.kodakarensis*, and very recently, *T.acidophilum*, to the best of our knowledge, this is the first report of an MCM helicase demonstrating DNA unwinding activity only at such acidic pH, across all archaea and eukaryotes. PtGINS may induce/stabilize a conducive conformation of PtMCM under acidic conditions, favouring PtMCM-mediated DNA unwinding coupled to ATP hydrolysis. Our findings underscore the existence of divergent modes of replication regulation among archaea and the importance of investigating replication events in more archaeal organisms.

DNA replication is broadly conserved across the three domains of life, beginning with the recognition of origins by initiator proteins, followed by the melting of origin DNA and stabilization of the single-stranded DNA thus formed. The two single strands of DNA serve as templates for the synthesis of the complementary strands. As DNA synthesis commences, the double-stranded DNA ahead of the advancing replication fork is unwound by replication-specific helicases. While DnaB is the replicative helicase in bacteria, the CMG (Cdc45 - MCM2-7 - GINS) complex is believed to be the active replicative helicase operating *in vivo* in eukaryotes (with MCM2-7 being the core helicase), and the MCM homohexamer is believed to be the archaeal replicative helicase (reviewed in Refs. 1, 2). DnaB is recruited to the origin by the helicase loader DnaC, while in eukaryotes the ORC, Cdt1 and Cdc6 facilitate the loading of the MCM2-7 helicase. No helicase loader has been clearly identified in archaea, though *in vitro* evidence suggests that the Orc1/Cdc6 protein may serve as both initiator and helicase loader^3^,^4^.

The eukaryotic MCM2-7 is a hexameric complex of six different but nevertheless closely related proteins that share a conserved domain of length ~200 amino acids, named the MCM box. First identified in *Saccharomyces cerevisiae* in a genetic screen for genes essential for chromosome maintenance^5^, these proteins are ubiquitously found from yeast to mammalian cells, and are a part of the pre-replication complexes (pre-RCs) that assemble at origins prior to replication initiation. The assembly of pre-RCs begins with the association of the ORC (**O**rigin **r**ecognition **c**omplex) with DNA, followed by the sequential recruitment of Cdc6, Cdt1 and MCM2-7, with ORC-Cdc6-mediated ATP hydrolysis facilitating the loading of the MCM heterohexamer. As cells enter S phase the MCM helicase is activated in a kinase-dependent manner. Thus, MCM2-7 is essential for both, initiation of DNA replication as well as progression of the replication fork (reviewed in Refs. 2, 6).

With archaeal DNA replication broadly resembling that of eukaryotes, the MCM helicase is among the several components of the replication apparatus that are conserved between eukaryotes and archaea. However, unlike the heterohexameric eukaryotic MCM2-7 helicase, the active archaeal helicase is typically a homohexamer (reviewed in Refs. 7, 8), with most archaea having a single MCM ortholog though some (like *Thermococcus kodakarensis)* have multiple MCMs^9^. The MCM homohexamer has been biochemically characterized in several archaea, including the euryarchaea *Methanothermobacter thermoautotrophicum, Archaeoglobus fulgidus, Pyrococcus furiosus, Thermococcus kodakarensis*, and *Thermoplasma acidophilum*, and the crenarchaea *Sulfolobus solfataricus* and *Aeropyrum pernix* (reviewed in Refs. 8, 10), and they typically exhibit 3′ to 5′ helicase activity *in vitro.*

The euryarchaeon *Picrophilus torridus* isolated from dry solfataric fields in Northern Japan thrives at temperatures of 55–60°C, and grows at an optimal pH of 0.7^11^,^12^. Annotation of its genome sequence revealed that it possesses characteristic euryarchaeal replication machinery comprising a single Orc1/Cdc6, a single MCM, a single-component GINS, and a homotrimeric PCNA^11^,^13^,^14^. PtOrc1/Cdc6 has been found to interact with PCNA; this interaction stimulates Orc1/Cdc6 ATPase activity in the presence of ORB-containing DNA, suggesting the possibility of the interaction playing a role in the regulation of DNA replication events^13^. The present study investigates the biochemical properties of the MCM protein of *P. torridus*. We find that PtMCM does not display significant helicase activity by itself *in vitro*, but rather, MCM helicase activity is drastically activated in presence of GINS. This activation of MCM helicase activity occurs at around pH 4, in keeping with the intracellular pH of *P. torridus.* Importantly, we find that at pH 4 GINS stimulates the ability of MCM to hydrolyze ATP, most dramatically at near physiological concentrations of ATP.

## Results

### *Picrophilus torridus* MCM is a homohexamer in solution and is expressed in logarithmically growing cells

**Figure 1:**
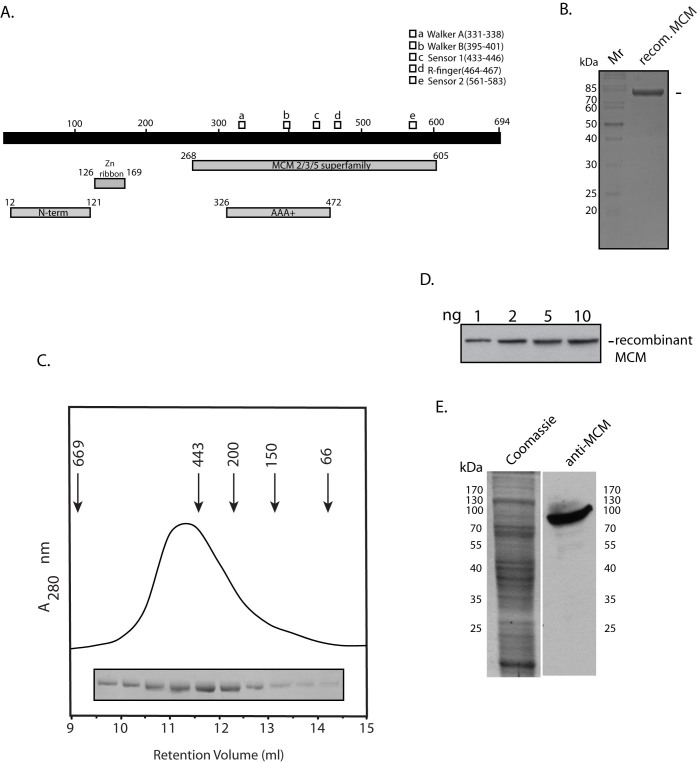
(A). Domain architecture of PtMCM. (B). SDS-PAGE (12%) analysis of purified recombinant PtMCM: Coomassie stain. Mr – molecular weight markers. Arrowhead indicates PtMCM. (C). Gel filtration analysis of PtMCM. Numbers with arrowheads indicate molecular weights and retention volumes of protein calibration markers. SDS-PAGE analysis of fractions matching the retention volumes are shown (full-length gel can be seen on-line in Supplementary Fig. S1A). (D). Western blot analysis of recombinant MCM protein with mouse anti-MCM antibodies (1:1000 dilution; full-length blot can be seen on-line in Supplementary Fig. S1B). (E). Western blot analysis of *Picrophilus* *torridus* extracts (6.5 × 10^7^ cell equivalents) with anti-MCM antibodies (1:1000 dilution). Coomassie stain shows the input extracts (1.6 × 10^7^ cell equivalents). Input extracts for Coomassie stain and for Western Blot were resolved on the same gel, the gel cut in half, one-half used for Western and other half stained with Coomassie.

The *MCM* gene annotated in the *Picrophilus torridus* whole genome sequence (PTO1217; KEGG Database) was amplified using its genomic DNA. The expected ~2.1 kb amplicon that was obtained was inserted into pUC19 and sequenced to confirm its veracity. Based on the derived amino acid sequence the protein was predicted to have a molecular weight of ~76 kDa. The sequence of the protein was analyzed using the Conserved Domains Database (CDD; www.ncbi.nlm.nih.gov/cdd), and the analysis predicted several domains typically found in archaeal MCM proteins, including the MCM2/3/5 superfamily domain (amino acids 268–605; Fig. 1A). The N-terminal domain found in most archaeal MCMs that comprises a bundle of four helices and an OB-like fold extends between residues 12–121, and the AAA+ (**A**TPase **a**ssociated with various **a**ctivities) domain carrying the Walker A and Walker B motifs that are essential for ATP binding and hydrolysis lies between residues 326–472. A zinc ribbon is predicted between amino acids 126–169.

PtMCM was expressed in *E.coli* with an N-terminal His tag (6xHis) and C-terminal Strep-tag (octapeptide sequence), and the recombinant protein of predicted monomeric molecular weight 81 kDa was purified to near homogeneity (Fig. 1B). To determine the molecular weight of the His-PtMCM-Strep protein, the purified protein (400 μg) was analyzed by gel filtration chromatography on a Superdex 200 column that had been calibrated with proteins of known molecular weight. As seen in Fig. 1C, PtMCM eluted just before apoferritin (Mol. Wt. 443 kDa). Thus, PtMCM exists predominantly as a hexamer in solution, in keeping with what has been reported in several other archaeal MCMs^15^,^16^,^17^. No peak corresponding to lower oligomeric/monomeric states was detected; however, the broad nature of the peak seen in Fig. 1C suggests that while the hexameric state is the predominant species, the protein might exist as a mixture of different oligomeric states in solution. The gel filtration elution profile of PtMCM did not change over the range of 1.5 to 5 μM protein. Polyclonal antibodies against PtMCM were raised in mice and rabbit, and were found to be sensitive enough to detect as little as 2 ng recombinant MCM at a 1:1000 dilution (Fig. 1D depicts Western Blot analysis using the mouse antibody). Western blot analysis of *P. torridus* whole cell extracts using these antibodies identified the presence of MCM in logarithmically growing cells (Fig. 1E).

### PtMCM-mediated ATP hydrolysis is DNA-independent and is dependent on the K337 residue

**Figure 2:**
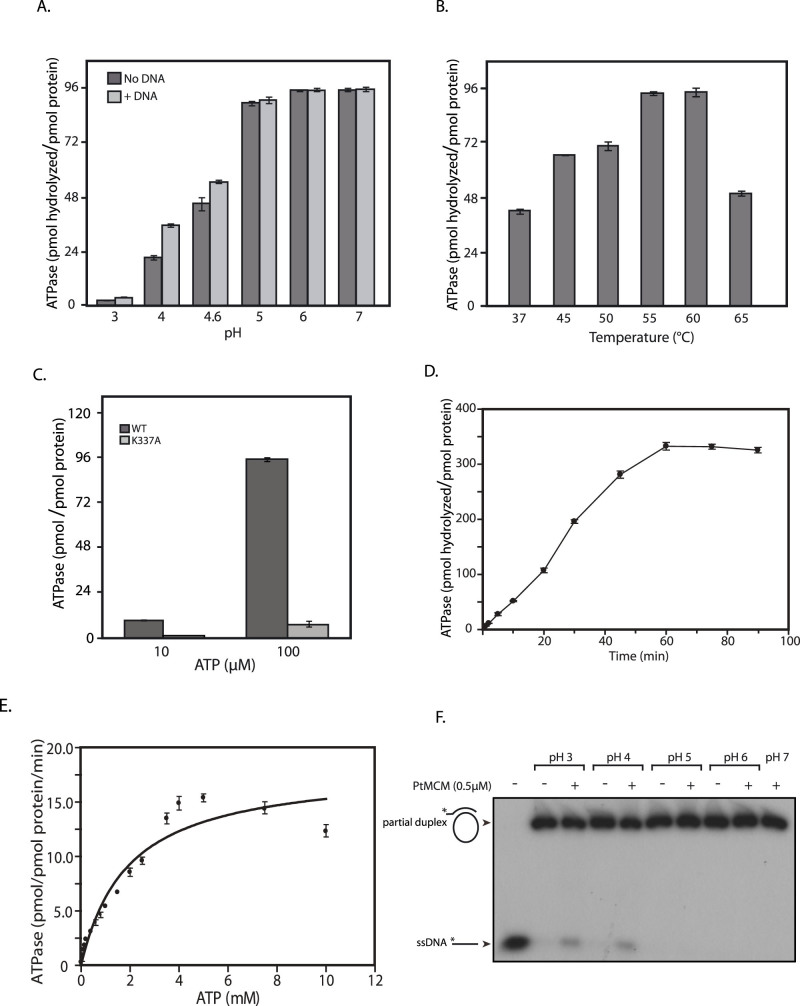
ATP hydrolysis assays. Data presented in (A–E) are the mean of three experiments and error bars represent standard deviation. (A). ATP hydrolysis as a function of pH. 800 nM PtMCM was incubated with 100 μM ATP in the presence or absence of DNA for 60 min. (B). ATP hydrolysis as a function of temperature. 800 nM PtMCM was incubated with 100 μM ATP in the absence of DNA at different temperatures for 60 min. (C). 800 nM PtMCM and PtMCM-K337A was incubated with 10 μM or 100 μM ATP in the absence of DNA for 60 min. (D). Time course of ATP hydrolysis reaction. 800 nM PtMCM was incubated with 1 mM ATP in the absence of DNA, and analysis carried out at different time points during the course of the reaction. (E). ATP hydrolysis as a function of ATP concentration. 800 nM PtMCM was incubated with 10–10,000 μM ATP in the absence of DNA for 25 min. (F). Helicase assay carried out in reactions of different pH using 500 nM PtMCM. Lane 1- radiolabelled ssDNA only (loaded as marker); lanes 2,4,6,8 – helicase reactions incubated in absence of protein; lanes 3,5,7,9,10 – helicase reactions incubated in presence of protein.

The hydrolysis of ATP is a key reaction in controlling the unwinding of DNA duplex by a helicase. To characterize the ability of PtMCM to hydrolyze ATP, protein purified by Strep-Tactin II affinity-based chromatography was further chromatographed on a Superdex 200 gel filtration column to exclude possible contaminating ATPases. We initiated the examination of PtMCM ATPase activity using the purified protein (800 nM) and ATP substrate [1000 pmoles (100 μM)], in reactions with and without DNA carried out in 30 mM Tris-acetate buffers of different pH values (ranging from 3 to 7). More than 70% of input ATP was hydrolyzed over pH 5–7 in 60 min, with the ability of PtMCM to hydrolyze ATP being clearly DNA-independent (Fig. 2A). To rule out the possibility of contaminating DNA in the protein preparations affecting the results obtained, the A_260_/A_280_ of purified MCM fractions were determined. Values ranged between 0.6 and 0.7, thus confirming that the protein preparation was free of DNA. While DNA had no effect on ATPase activity at pH 5–7, at pH 4 the presence of DNA in the reaction stimulated ATPase activity about two-fold (Fig. 2A). All assays thereafter were performed at pH 6, and at 55°C as the ATP hydrolysis reaction was equally efficient at 55°C and 60°C (in keeping with the normal growth temperature of the organism; Fig. 2B). When we analyzed ATPase activity as a function of PtMCM concentration ranging over 10–1200 nM protein, we found almost 80% of the input 1000 pmoles ATP was hydrolyzed by 1000 nM protein in 60 min (Fig. S2A).

The Walker A motif of members of the AAA+ family carries a conserved lysine residue that is critical to ATP binding. The conserved lysine residue in the PtMCM Walker A motif (K337) was mutated to alanine, and the recombinant mutant protein PtMCM-K337A was purified for ATPase assays using the same procedure as was used for wild type protein. As evident from Fig. 2C, ATPase activity was drastically diminished in case of the mutant PtMCM-K337A. The progress of the ATP hydrolysis reaction by PtMCM was monitored over a period of 90 min and the reaction was found to move forward almost linearly upto 45 min before plateauing at around 60 min (Fig. 2D). On investigating the ability of PtMCM (800 nM) to hydrolyze different concentrations of input ATP, it was observed that more than 1800 pmoles of ATP were hydrolyzed in 25 min when 2.5 mM ATP was added to the reaction, with PtMCM displaying a specific activity of approximately 14.5 pmol pmol^−1^min^−1^ (pmoles ATP hydrolyzed per pmole protein per minute) at 7.5 mM ATP substrate concentration (ATP titration versus 800 nM PtMCM was performed thrice and the mean values are presented in Fig. 2E, with error bars representing standard deviation). Using GraphPad Prism 4.0 we determined the K_m_ to be 1.2 mM (in the same range as that of AfMCM and ApeMCM^15^,^18^). These data collectively demonstrate that PtMCM exhibits a dynamic ability to hydrolyze ATP and the conserved lysine K337 in its Walker A motif is essential for this.

### Cloning of PtGINS and its expression in *Picrophilus torridus*

The archaeal MCM homohexamers (or dodecamers) characteristically display helicase activity without the need for auxiliary factors *in vitro*. We began investigations of the PtMCM helicase activity using the purified homohexamer and a forked substrate that was M13-derived (Fig. 2F), in 30 mM Tris-acetate buffer as described in Methods. However, although we tried a wide range of conditions including varying protein concentrations, ATP concentrations, temperature and salt and magnesium ion concentrations, we were unable to detect a robust helicase activity over pH range 3–7, with only weak activity being detected at pH 3 and pH 4 and none being detected at pH 5–7 (Fig. 2F and Fig. S2B). This was somewhat surprising, as PtMCM exhibited 2.5 to 3.5 -fold more ATPase activity at pH 5–7 than at pH 3–4 when 100 μM ATP substrate was used (Fig. 2A). While most archaeal MCMs by themselves exhibit helicase activity, in some euryarchaeal microbes such as *Pyrococcus furiosus, Thermococcus kodakarensis and Thermoplasma acidophilum* the GINS protein has been found to stimulate MCM helicase activity^9^,^14^,^19^. As we were unable to detect a robust MCM helicase activity in spite of our best efforts, we explored the possibility of GINS being essential for activating PtMCM helicase activity.

**Figure 3:**
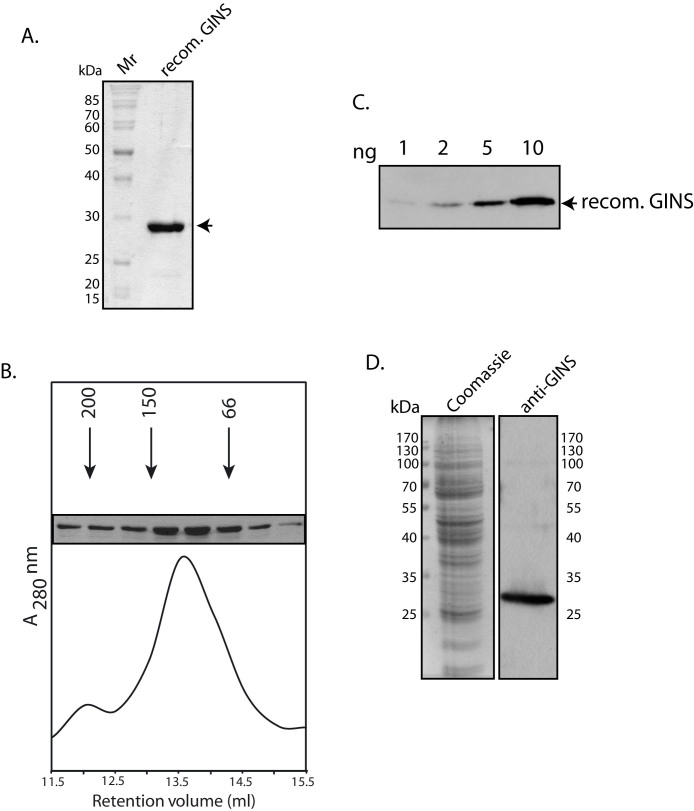
(A). SDS-PAGE (10% PAGE) analysis of recombinant GINS – Coomassie stain. Mr – molecular weight markers. Arrowhead indicates PtGINS. (B). Gel filtration analysis of PtGINS. Numbers with arrowheads indicate molecular weights and retention volumes of protein calibration markers. SDS-PAGE analysis of fractions matching the retention volumes are shown (full-length gel can be seen on-line in Supplementary Fig. S3A). (C). Western blot analysis of recombinant GINS protein with mouse anti-GINS antibodies (1:1000 dilution; Full-length blot can be seen on-line in Supplementary Fig. S3B). (D). Western blot analysis of *Picrophilus* *torridus* extracts (6.5 × 10^7^ cell equivalents) with anti-GINS antibodies (1:1000 dilution). Coomassie stain shows the input extracts (1.6 × 10^7^ cell equivalents). Input extracts for Coomassie stain and for Western Blot were resolved on the same gel, the gel cut in half, one-half used for Western and other half stained with Coomassie.

While the eukaryotic GINS is a heterotetramer, the archaeal GINS is a simpler protein, either made of two kinds of subunits and existing as a dimer of dimers as in *S. solfataricus* and *P. furiosus*^14^,^20^, or made of one subunit and existing as a homotetramer as in *T. acidophilum*^21^. Based on whole genome sequence analyses, *P. torridus* has a single GINS ortholog (PTO0266^14^). The gene encoding PtGINS was cloned and the recombinant protein expressed in *E. coli* with an N-terminal His-tag (monomeric molecular weight ~ 25 kDa). The protein was purified to near homogeneity (Fig. 3A), and its oligomeric state determined by chromatography on Superdex 200 (Fig. 3B). By using the retention volumes of the standard marker proteins, it was determined that PtGINS exists as a tetramer, similar to what is reported in *T. acidophilum*. The antibodies raised against PtGINS in mice could detect upto 2 ng recombinant protein (Fig. 3C), and Western blot analysis of *Picrophilus* whole cell extracts using these antibodies revealed the presence of the protein in logarithmically growing cells (Fig. 3D).

### GINS associates stably with MCM in *Picrophilus* extracts, and interacts with MCM directly *in vitro*

**Figure 4:**
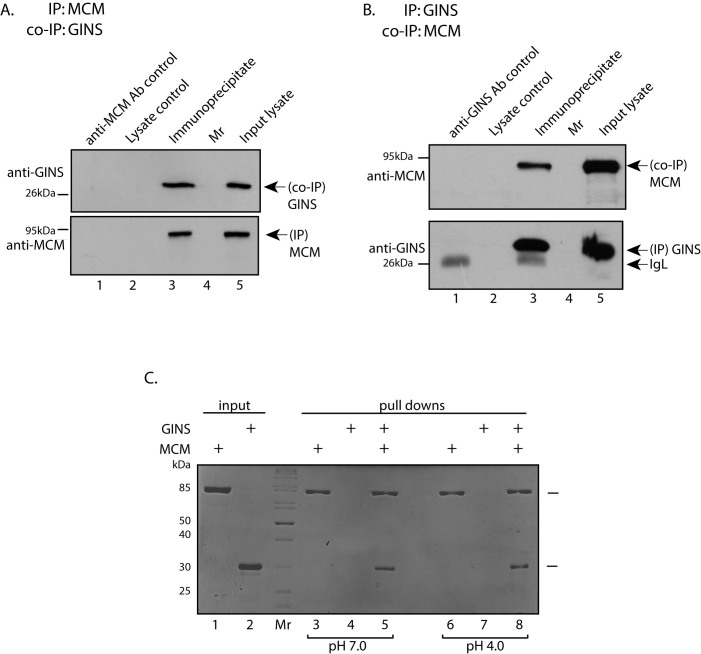
(A & B). Immunoprecipitation analysis. (A): PtMCM immunoprecipitates were resolved by SDS-PAGE (10%) and analyzed by western blotting using anti-MCM (to detect immunoprecipitated MCM) or anti-GINS (to detect co-immunoprecipitating GINS) antibodies (1:1000 dilution). Full-length blots can be seen on-line in Supplementary Fig. S4A. (B): PtGINS immunoprecipitates were similarly analyzed. Full-length blots can be seen on-line in Supplementary Fig. S4B. Lanes 1: antibody control (bead-bound antibodies only and no lysate added); lanes 2: lysate incubated with beads only (with no antibody coupled to the beads); lanes 3: immunoprecipitation reaction; lanes 4: molecular weight marker; lanes 5: input lysate. (C). MCM pulldown experiment: Coomassie stain of SDS-PAGE analysis. Lane 1-input purified PtMCM; lane 2 – input purified PtGINS; lanes 3 to 8 – analysis of eluate fractions of pulldowns. Lanes 3 and 6 - recombinant MCM alone incubated with Strep -Tactin Superflow affinity beads; lanes 4 and 7- recombinant GINS alone incubated with Strep -Tactin Superflow affinity beads; lanes 5 and 8- recombinant MCM plus recombinant GINS incubated with Strep-Tactin Superflow affinity beads. Mr – molecular weight marker.

We investigated if GINS and MCM complexed with each other in *Picrophilus* extracts by carrying out immunoprecipitation reactions and analyzing the immunoprecipitates for co-immunoprecipitating protein. Accordingly, MCM was immunoprecipitated from isolated *P. torridus* extracts with rabbit anti-MCM antibody and immunoprecipitates analyzed, as described earlier^13^. Western blot analysis of the immunoprecipitates that had been resolved by SDS-PAGE revealed that GINS interacted with MCM in these extracts (Fig. 4A). The converse experiment, where GINS was immunoprecipitated from whole cell lysates using mouse anti-GINS antibody and the immunoprecipitate analyzed for co-immunoprecipitating MCM, confirmed that MCM and GINS associate with each other in *Picrophilus* extracts (Fig. 4B), though perhaps not directly.

Direct pulldown experiments using the two recombinant proteins were carried out to determine if the association was direct. As seen in Fig. 4C, at both pH 4 and pH 7, GINS was pulled down along with MCM using Strep-Tactin affinity beads (lanes 5 and 8 respectively). GINS was not pulled down in absence of MCM (Fig. 4C lanes 4 and 7). Thus, having ascertained that GINS interacted with MCM in cell extracts, and directly bound to MCM *in vitro*, we examined MCM helicase activity in presence of GINS.

### PtMCM displays helicase activity in the presence of PtGINS in a pH-dependent manner

**Figure 5:**
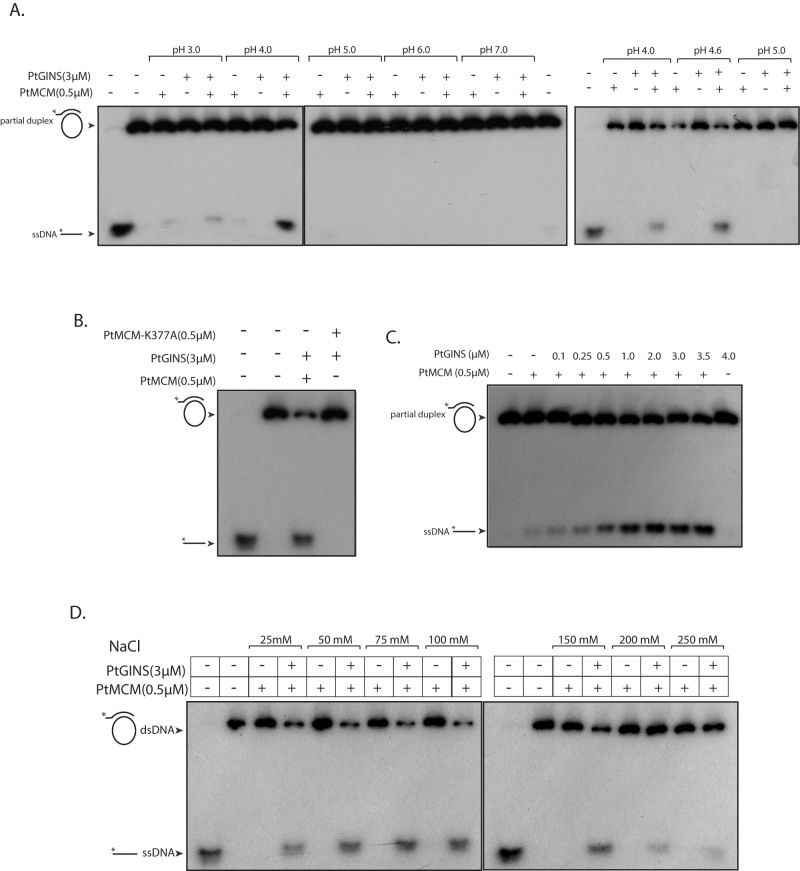
Helicase assays. (A). Reactions carried out with 500 nM PtMCM in presence and absence of PtGINS (3000 nM) in 30 mM Tris-acetate buffers of different pH. Reactions were analyzed on three separate 6% gels, and run under identical conditions of voltage. Cropping lines indicate separate gels. Lane 1 of first and last gels - radiolabelled ssDNA only (loaded as marker); lane 2 of first gel and last lane of second gel– helicase reactions incubated in absence of protein (B). Reactions carried out with 500 nM PtMCM (wild type and K337A mutant) and 3000 nM PtGINS in 30 mM Tris-acetate buffer of pH 4. Lane 1- radiolabelled ssDNA only (loaded as marker); lane 2 – helicase reaction incubated in absence of protein (C). Reactions carried out with 500 nM MCM and increasing concentrations of GINS in 30 mM Tris-acetate buffer of pH 4. Lane 1 – helicase reaction incubated in absence of protein (D). Reactions carried out with 500 nM PtMCM in presence and absence of 3000 nM GINS in 30 mM Tris-acetate buffer of pH 4; NaCl concentrations in the reaction varied from 25 mM to 250 mM. Reactions were analyzed on two separate 6% gels, and run under identical conditions of voltage. Cropping lines indicate separate gels. Lanes 1 of both gels- radiolabelled ssDNA only (loaded as marker); lanes 2 of both gels – helicase reaction incubated in absence of protein. All reactions were performed at 55°C for 1 h. Composition of helicase assay buffers detailed in Methods.

PtMCM was analyzed for helicase activity in the presence of GINS, in 30 mM Tris-acetate buffer of pH ranging between 3 to 7. For this, GINS purified by TALON metal affinity chromatography was also further subjected to gel filtration chromatography to minimize chances of contaminating *E. coli* helicases. While very weak activity was detected in absence of GINS at pH 3 and pH 4, activity dramatically increased at pH 4 and pH 4.6 when GINS was added to the reaction (Fig. 5A). No activity was apparent at pH 5–7. To the best of our knowledge, this is the first MCM helicase to display helicase activity *in vitro* only at such an acidic pH. We attributed the detected helicase activity to MCM and not to any contaminating *E. coli* helicase activity co-purifying with MCM because the protein that had been purified by affinity based chromatography was further subjected to gel filtration chromatography before using it in helicase assays, and more importantly, because replacing the wild-type MCM with the MCM-K337A mutant in helicase assays resulted in lack of helicase activity (Fig. 5B) although MCM-K337A retained its ability to interact with GINS at both pH 4 and pH 7 in direct pulldown experiments (Fig. S5). GINS activated MCM helicase function in a concentration-dependent manner over 250–3500 nM GINS (using 500 nM MCM; Fig. 5C), and increasing MCM concentrations upto 2 μM did not particularly enhance MCM helicase activity in absence of GINS (data not shown). The ability of PtMCM to unwind duplex DNA was examined under different salt conditions at pH 4.0, in the presence and absence of GINS. We found that helicase activity was detected using sodium chloride concentrations varying from 25 mM to 250 mM, with optimum activity being apparent at 50–150 mM sodium chloride, and in all cases MCM-mediated DNA unwinding was activated by GINS (Fig. 5D).

Considering that the strength of the interaction between MCM and GINS is comparable at pH 4 and pH 7 *in vitro* (Fig. 4C), and GINS interacts with MCM in extracts that have been isolated at neutral pH (Fig. 4A, 4B), it is possible that the GINS-MCM interaction results in MCM adopting a strong kinetically favourable conformation for DNA unwinding activity only around the physiological pH of *Picrophilus*.

### PtOrc1/Cdc6 does not significantly influence PtMCM helicase or ATPase activity

**Figure 6:**
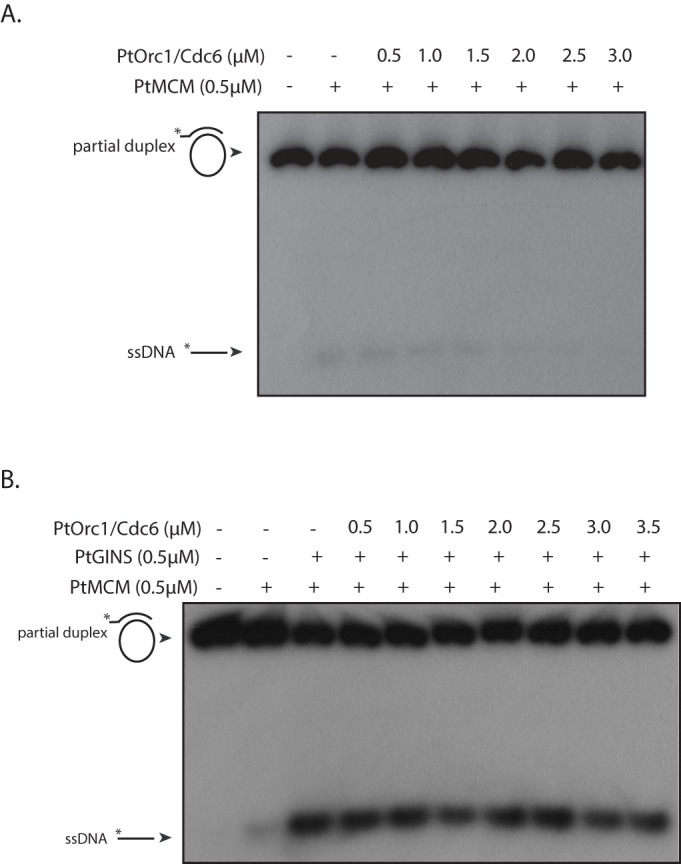
(A). Helicase assay carried out with 500 nM PtMCM in presence of increasing concentrations of PtOrc1/Cdc6, in 30 mM Tris-acetate buffer of pH 4. (B). Helicase assay carried out with 500 nM PtMCM in presence of 500 nM GINS and increasing concentrations of PtOrc1/Cdc6, in 30 mM Tris-acetate buffer of pH 4. Lanes 1 of both gels – helicase reaction incubated in absence of protein.

Archaeal Orc1/Cdc6 proteins do not have a typical impact on the ability of archaeal MCM proteins to unwind DNA. While Orc1/Cdc6 enhances MCM-mediated helicase activity in some cases^17^,^22^, in other cases it inhibits the helicase activity of MCM^15^,^23^,^24^. We examined the effect of PtOrc1/Cdc6 (purified as described^13^) on PtMCM helicase activity and found that the weak activity displayed by PtMCM was not stimulated by PtOrc1/Cdc6 over a concentration range of 0.5 to 3 μM Orc1/Cdc6 (Fig. 6A). To see the effect of PtOrc1/Cdc6 on helicase activity of PtMCM in the presence of GINS, 0.5 to 3 μM PtOrc1/Cdc6 was added to helicase reactions carrying 0.5 μM each of PtMCM and PtGINS. No significant effect of Orc1/Cdc6 was apparent (Fig. 6B). We also examined the effect of PtOrc1/Cdc6 on PtMCM ATPase activity. For this, different concentrations of PtOrc1/Cdc6-K67A (ATPase-dead mutant^13^) were added to ATP hydrolysis reactions with PtMCM in absence and presence of DNA. We found that PtOrc1/Cdc6 did not have any effect on ATPase activity of PtMCM (Fig. S6).

### GINS does not appear to modulate the ability of PtMCM to bind DNA

**Figure 7:**
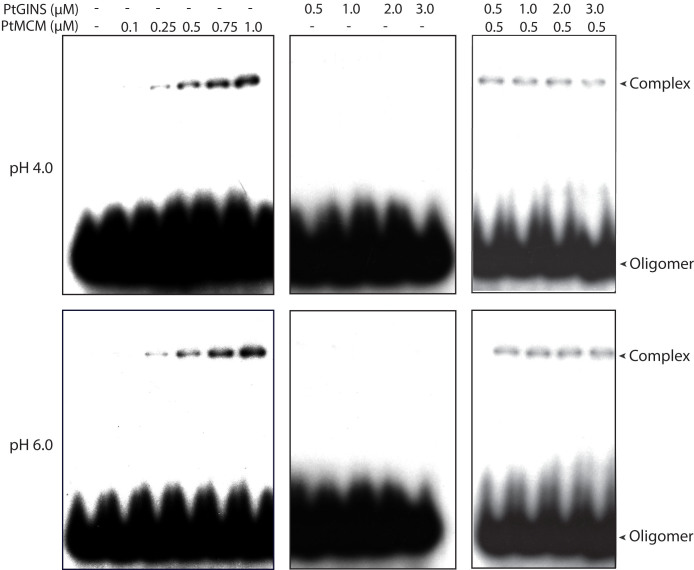
Electrophoretic mobility shift assays carried out in buffer of pH 4 or pH 6, using either MCM alone (0.1 to 1 μM), or GINS alone (0.5 to 3 μM), or MCM (0.5 μM) and increasing concentrations of GINS (0.5 to 3 μM). Double-stranded forked DNA was used as substrate.

To investigate the likely means by which GINS may stimulate MCM helicase activity, and then too only at acidic pH, we first studied the DNA binding properties of PtMCM at pH 4 and pH 6 in absence and presence of GINS using EMSAs. For this, forked dsDNA was used as substrate. Protein-DNA complexes were detected when PtMCM was incubated with the substrate (as described in Methods) in protein concentration-dependent manner (Fig. 7, left panels of both rows). Amounts of complexes detected at both pHs were more or less comparable, though we were unable to quantitate the ratios of bound to unbound substrate as the percentage of detectable binding was very low. The addition of GINS to the reactions did not have any discernible impact on the amount/nature of complexes detected (Fig. 7, right panels of both rows). No complexes were detected when GINS alone was incubated with the substrate (Fig. 7, centre panels of both rows). We also tested the interaction of PtMCM with ssDNA, and were unable to detect any binding of PtMCM to the substrate (data not shown). Our data suggest that the interaction of PtMCM with its substrate DNA does not vary between pH 4 and pH 6 and is not affected by PtGINS, though it is important to keep in mind that the binding reactions are resolved on gels run in 1X TBE (neutral pH) and we may only be detecting those complexes which are stable under these electrophoresis conditions.

### GINS stimulates the ability of MCM to hydrolyze ATP at pH 4 but not at near-neutral pH

**Figure 8:**
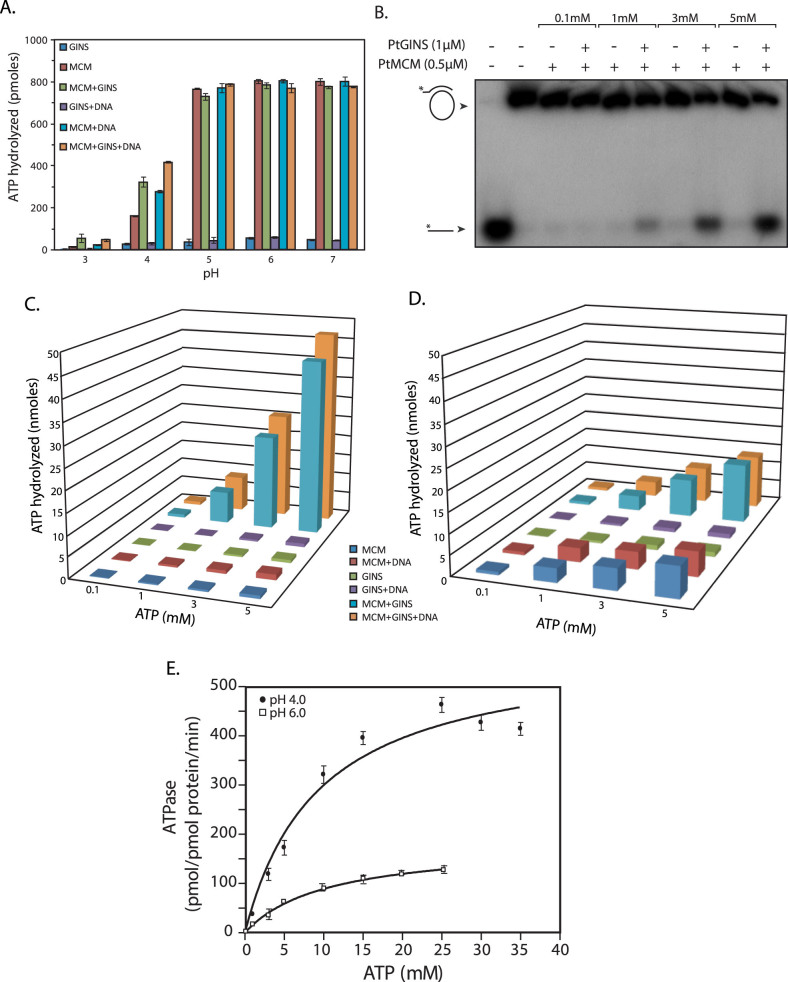
(A). ATP hydrolysis assay in reactions of different pH carried out in presence or absence of GINS. 500 nM PtMCM and 3000 nM PtGINS were used in reactions containing 100 μM ATP, carried out in presence or absence of DNA for 60 min. (B). Helicase assays carried out using 500 nM PtMCM and 3000 nM PtGINS with varying concentrations of ATP (reaction at pH 4). Lane 1- radiolabelled ssDNA only (loaded as marker); lane 2 – helicase reaction incubated in absence of protein (C). Amount of ATP hydrolyzed during the helicase reactions carried out with varying concentrations of ATP using 500 nM PtMCM and 3000 nM PtGINS (reaction at pH 4 for 60 min). (D). Amount of ATP hydrolyzed during the helicase reactions carried out with varying concentrations of ATP using 500 nM PtMCM and 3000 nM PtGINS (reaction at pH 6 for 60 min). (E). ATP hydrolysis as a function of ATP concentration. 500 nM PtMCM and 3000 nM PtGINS was incubated with 0.1 to 35 mM ATP in the absence of DNA for 25 min. Data presented in (A), (C), (D) and (E) are the mean of three experiments and error bars represent standard deviation.

The effect of the MCM-GINS interaction on the ability of MCM to hydrolyze ATP was examined next. We first assessed this in the presence and absence of DNA at pH ranging over 3–7 using 100 μM ATP. As was seen earlier (in Fig. 2), significant amounts of ATP were hydrolyzed by MCM alone at pH 4 and above, with highest amounts of hydrolysis at pH 5–7, and this hydrolysis was DNA-independent (Fig. 8A). DNA had no effect on ATP hydrolysis at pH 5–7, but ATP hydrolysis was stimulated by the addition of DNA at pH 4. Interestingly, when GINS was added to the reaction along with MCM, it had no effect on ATP hydrolysis at pH 5–7, but at lower pH ATP hydrolysis increased almost two-fold.

To determine the amount of ATP being hydrolyzed by MCM during the helicase reaction in presence and absence of GINS, we analyzed the helicase reaction carried out at pH 4 at different ATP concentrations, for DNA unwinding activity as well as ATP hydrolysis activity. For this, we performed the helicase assay using radiolabelled DNA substrate (2.5 nM) with different concentrations of ATP, and with non-radiolabelled DNA substrate (2.5 nM) using different concentrations of cold ATP and radiolabeled ATP as tracer, in parallel reactions. Assays performed with radiolabelled DNA substrate were analyzed for DNA unwinding activity by PAGE, and activity found to be negligible at 100 μM ATP and maximum at 5 mM ATP (Fig. 8B). Assays performed with non-radiolabelled DNA substrate and radiolabelled ATP (tracer) were analyzed for ATP hydrolysis activity by TLC as earlier (Fig. 8C). For ATP hydrolysis analyses, identical parallel reactions were also set up in the absence of the helicase DNA substrate. As seen in Fig. 8C, the presence of GINS in the reaction stimulated ATP hydrolysis more than 50-fold at 5 mM ATP concentration, regardless of the presence of DNA in the reaction.

Considering the apparently contradictory facts that no helicase activity was apparent over pH 5–7 (Fig. 5A), while ATP hydrolysis appeared to be optimal at pH 5–7 when 100 μM ATP was used as substrate (Fig. 2A, 8A), we analyzed the amount of ATP hydrolyzed during helicase reactions carried out using different ATP concentrations at pH 6 as described above. Comparing the data obtained at pH 4 with that obtained at pH 6 (Fig. 8C versus Fig. 8D), we found that in the absence of GINS, MCM-mediated ATP hydrolysis was substantially higher at pH 6 than at pH 4, both in the presence and absence of DNA. However, while GINS stimulated MCM ATPase activity more than fifty-fold at pH 4 at 3–5 mM ATP, the effect of GINS on MCM ATPase activity was modest at pH 6 at the same ATP concentrations, with about two-fold increase in activity. To examine if GINS altered the binding affinity of MCM for ATP more favourably at pH 4 compared to at pH 6, we determined the K_m_ for ATP in the presence of GINS at both pH 4 and pH 6. As seen in Fig. 8E, the addition of GINS increased the K_m_ approximately four-fold at pH 6, from 1.2 mM in the absence of GINS (Fig. 2E) to 5.1 mM. This apparent decrease in binding affinity was compensated for by an overall increase in catalytic efficiency upon the addition of GINS (an ~ eight-fold increase in V_max_ seen in Fig. 8E compared to Fig. 2E). We found the K_m_ for ATP in the presence of GINS at pH 4 also to be around 5 mM (Fig. 8E), signifying that the binding affinity for ATP was comparable at both pHs. However, the catalytic efficiency of the enzyme was considerably higher at pH 4 compared to pH 6 (compare V_max_ at pH 4 versus at pH 6).

## Discussion

Organisms belonging to the third domain of life (Archaea) thrive in a wide range of extreme environmental conditions including high temperatures, low pH, high salinity etc. The metabolic pathways in these organisms typically resemble those found in bacteria, but the information processing machinery including those for DNA replication, transcription and translation are comparable with what is found in eukaryotes. The present study was undertaken to work towards understanding DNA replication events in the extremophile *Picrophilus torridus*, and targets the MCM protein.

Structure-function relationships have been well studied in MCM from *M. thermautotrophicus* and *S. solfataricus*^25^,^26^,^27^,^28^,^29^,^30^,^31^,^32^,^33^,^34^,^35^,^36^,^37^,^38^,^39^. Typically, archaeal MCM proteins are between 625 to 700 amino acids in length, and are broadly structured into three domains – an N-terminal region that is devoid of catalytic activity but is important for DNA binding, MCM hexamerization, as well as regulation of helicase activity; a central catalytic region that carries the motifs characteristic of all members of the AAA+ family of ATPases; and a C-terminal region containing a helix-turn-helix domain. Detailed domain-mapping studies will have to be carried out to ascertain the functional roles of the predicted N-terminal domain and central catalytic domain of PtMCM protein. Biochemical characterization of PtMCM revealed that while it shared some properties with other archaeal MCMs, it behaved distinctly in other ways. Although dodecamers and heptamers have been reported in case of *M*. *thermautotrophicus* MCM^28^,^29^,^31^,^40^,^41^,^42^ most archaeal MCMs are homohexamers^9^,^15^,^16^,^17^,^43^, as is PtMCM. Archaeal MCMs have shown variable ATPase activities. PtMCM behaved like SsoMCM, TaMCM, PfMCM, ApeMCM, AfMCM and TkMCMs, which display the ability to hydrolyze ATP regardless of the presence/absence of DNA in the reaction (although in some cases like AfMCM, ApeMCM and TkMCMs DNA stimulates the activity), unlike MthMCM which is a DNA-dependent ATPase^9^,^14^,^15^,^16^,^18^,^40^,^42^,^44^.

The first report characterizing an archaeal MCM demonstrated that the *M. thermautotrophicus* MCM possessed 3′ to 5′ helicase activity *in vitro* without the necessity of ancillary proteins^45^. Subsequently several reports from different archaea demonstrated that the MCM homohexamer/dodecamer possessed an independent ability to unwind DNA^9^,^16^,^18^,^40^,^42^,^44^,^46^. Our results indicate that PtMCM needs GINS as an auxiliary factor. First identified in *S. cerevisiae* as essential for replication^47^, the eukaryotic GINS exists as part of the CMG (Cdc45, MCM, GINS) complex responsible for unwinding the DNA duplex so that replication may occur (reviewed in Ref. 48). The archaeal GINS was first identified in the hyperthermophilic acidophile *Sulfolobus solfataricus* by computational analysis^49^, and as an interacting partner of the MCM protein in a yeast two hybrid screen^20^. However, this interaction had no apparent effect on MCM activity. Although PtGINS interacted directly with PtMCM in pull down assays the MCM-GINS interaction was not stable enough to be detected in gel filtration analyses of reactions in which MCM and GINS were co-incubated, either in presence or absence of DNA (analysis by monitoring A_280_ as well as by SDS-PAGE analysis of eluate fractions in absence of DNA, and by SDS-PAGE analysis of eluate fractions in presence of DNA; data not shown).

The effect of PtGINS on PtMCM helicase activity was very pronounced and pH-specific. MCM helicases of other acidophiles such as *S. solfataricus* and *T. acidophilum* have been characterized, and although these two organisms grow at optimum pH of 3.5 and 2.0 respectively, their MCMs exhibit optimal helicase activity at pH 7.5 to 8.5^16^,^44^. The difference in the behavior of PtMCM compared to SsoMCM or TaMCM may be attributed to the fact that *S. solfataricus* and *T. acidophilum* both maintain intracellular pH close to neutral (6.5 and 5.5 respectively), while the extremely acidophilic *P. torridus* that grows at optimum pH 0.7 maintains an intracellular pH of approximately 4.6^50^. Thus, PtMCM appears to be active only under conditions that match the organism's physiological pH. It is, however, surprising that we have been unable to detect any helicase activity at pH 5 in spite of extensive efforts. The reasons for this are not understood at this time.

The helicase reaction involves a series of three main steps – loading of helicase onto DNA substrate, translocation of helicase along the unwinding DNA that is driven by ATP hydrolysis, and release of DNA product from the enzyme (enzyme turnover). ATPase activity of PtMCM by itself is higher at around neutral pH than acidic pH (Figs. 2A, 8A, 8C and 8D), yet helicase activity of PtMCM by itself (although weak) is detected only at around pH 4. This indicates that the ATPase rate is not the cause of lack of detectable helicase activity at pH 6. Considering the possibility of the loading of MCM onto the DNA substrate being favoured at around pH 4 compared to higher pH, the data from the EMSAs suggest that this may not be so as the protein is loaded comparably at both pHs (Fig. 7), although we may be detecting only those complexes which are stable under 1X TBE run conditions. Taken together, these data suggest that the rate-limiting step that is controlling the overall kinetics of the helicase reaction at pH 6 versus pH 4, is the last step (release of DNA product from enzyme), though the enzyme loading step (onto DNA substrate) cannot be completely ruled out. MCM may adopt a kinetically more favourable conformation for this step at pH 4 as compared to higher pH, resulting in very poor turnover (MCM hexamer release from DNA) at higher pH. Detailed structure-function analyses needs to be carried out to ascertain the mechanism by which this is occurring.

The helicase activity is strongly enhanced in the presence of GINS at around pH 4 but not at higher pH. The results from the EMSA experiments suggest that GINS does not significantly impact the loading of MCM onto DNA substrate, or help stabilize MCM-DNA complex, though again one must consider the fact that we are detecting only those complexes that are stable under the electrophoresis run conditions. ATPase activity is enhanced about 50-fold by GINS at pH 4 but only about two-fold at higher pH, at near-physiological concentrations of ATP (Figs. 8C, 8D). In the presence of GINS, MCM's binding affinity for ATP is comparable at pH 4 and pH 6, however, the overall catalytic efficiency of the helicase is much higher at pH 4 (Fig. 8E). Therefore we conclude that GINS mediates its effect on MCM helicase activity largely by activation of MCM's ability to hydrolyze ATP, increasing its ability to generate energy to drive the DNA unwinding reaction efficiently. However, this may not be the only mechanism by which GINS exerts its influence. The strength of the MCM-GINS interaction appears to be comparable at pH 4 and pH 6 (Fig. 4), and we speculate that GINS may support the adoption/stabilization of a kinetically more favourable conformation by MCM at pH 4, which promotes both ATP hydrolysis as well as release of DNA product. Future studies will be directed towards unravelling the molecular mechanism by which GINS impacts MCM activity.

In conclusion, *Picrophilus torridus* is now one of four archaeal species whose MCM homohexamer's ability to unwind DNA is activated by GINS. All four are euryarchaeal organisms; however, MCM from other euryarchaeal species such as *A. fulgidus* and *M. thermoautotrophicum* demonstrate independent helicase activity. Thus, our findings underline the fact that there is diversity in modes of replication among archaea, and even among euryarchaea, and emphasize the importance of investigating DNA replication in more members of the third domain.

## Methods

### *Picrophilus torridus* cultures and lysates

*Picrophilus torridus* DSM 9790 was grown in liquid medium under aerobic conditions as described earlier^13^. For analysis of expression of PtMCM and PtGINS, logarithmically growing cells were harvested, washed with PBS, and directly lysed in SDS-PAGE sample loading dye. For carrying out immunoprecipitations, whole cell extracts were isolated as described earlier^13^.

### Cloning of genes encoding MCM and GINS

The genes encoding MCM and GINS were cloned from *P. torridus* genomic DNA by amplification using end primers whose sequences were derived from the respective genes annotated in the *P. torridus* whole genome sequence^11^,^14^. PtMCM Walker A mutant (*PtMCM-K337A)* was created by overlapping PCR. *PtMCM* and *PtMCM-K337A* were independently subcloned from their pUC clones into the BamHI-SalI sites of vector pASK-IBA43plus (IBA BioTAGnology, Germany), creating plasmids pASK-MCM and pASK-MCM-K337A for expression in *E. coli. PtGINS* was expressed in *E. coli* by subcloning the gene from pUC-GINS into the BamHI-SalI sites of pET-28a, creating plasmid pET-GINS.

### Purification of PtMCM and PtGINS

The recombinant PtMCM proteins tagged with six histidine residues at the N-terminus and a Strep-tag (octapeptide) at the C-terminus were expressed in soluble form at 16°C in *E.coli* BL21 Codon Plus cells, and were purified to near homogeneity by successive chromatography on TALON metal affinity resin (BD Biosciences) and Strep-Tactin II affinity-based chromatography (IBA BioTAGnology, Germany) as per the manufacturers' protocols. The recombinant PtGINS tagged with six histidine residues at the N-terminus was expressed at 16°C, and the soluble protein was purified by affinity-based chromatography using TALON metal affinity resin (BD Biosciences).

### Raising antibodies to PtMCM and PtGINS

Polyclonal antibodies against the purified proteins were raised in mice as described earlier^13^. Antibodies against PtMCM were also raised in rabbit. For this, the primary immunization was carried out with 500 μg of purified MCM protein along with Freund's complete adjuvant. Subsequent booster doses were given using the same amount of protein with Freund's incomplete adjuvant. The rabbit was bled ten days after the final booster shot.

### Immunoprecipitations and pull downs

Immunoprecipitations were carried out using antibodies coupled to Protein A-sepharose beads (Sigma Aldrich, USA). Antibodies were immobilized by incubating 5 μl antibodies with a protein A sepharose/CL6B sepharose 1:1 slurry in 1X PBS at 4°C for 90 min, with periodic mixing at 5–10 min intervals. The beads were washed with 1X PBS/0.2% Triton X-100 to remove unbound antibody. MCM or GINS was immunoprecipitated from lysates of cells that had been harvested from 100 ml cultures grown to O.D._600_ ~ 0.4. PtMCM was immunoprecipitated using immobilized rabbit anti-MCM antibodies while PtGINS was immunoprecipitated using immobilized mouse anti-GINS antibodies. Immunoprecipitates were isolated and analyzed as detailed earlier^13^.

Pull down assays were performed by first incubating purified His-MCM-Strep and His-GINS proteins together, either in 500 μl 1X PBS, or in 100 mM Tris-acetate buffer (pH 4.0) containing 150 mM NaCl, with mixing for 30 min at 4°C using a nutator mixer. MCM (and any GINS that was interacting with it) was pulled down from the reaction using Strep-Tactin Superflow affinity beads (IBA BioTAGnology), by incubating the interaction reaction mix with the beads for 15 min at 4°C. The unbound fraction was removed and the beads were washed extensively (with 100 mM Tris-Cl (pH 7.0), 150 mM NaCl for analyzing interaction at pH 7; or with 100 mM Tris-acetate buffer (pH 4.0) containing 150 mM NaCl for analyzing interaction at pH 4). The bound MCM (and any interacting GINS) was eluted, and the eluates were analyzed by resolution on SDS-PAGE followed by Coomassie staining.

### Gel filtration analysis

Gel filtration analysis was performed using a Superdex 200 10/300 GL analytical column (GE Healthcare Life Sciences) coupled to the AKTA FPLC system (GE Healthcare Life Sciences) as described earlier^13^. Briefly, for purifying proteins for biochemical assays, protein eluted from Strep-Tactin II resin or TALON beads were resolved chromatographically on Superdex 200 in 50 mM potassium phosphate (pH 7.4), 150 mM NaCl. For determining the molecular weight of PtMCM and PtGINS the column was calibrated with protein standards of known molecular weights (obtained from Sigma Aldrich). The molecular weights of PtMCM and PtGINS were determined from their retention volumes, by comparison with those of the standards.

### ATP hydrolysis assay

PtMCM protein purified by TALON metal affinity chromatography followed by Strep-Tactin II affinity-based chromatography (as described above) was further loaded on Superdex 200 (GE Healthcare) and subjected to gel filtration chromatography. Eluate fractions were analyzed by SDS-PAGE, and the concentrations of the eluted PtMCM protein estimated using Bradford's method (Bio-Rad Protein Assay Kit), for use in ATP hydrolysis assays. Typically, 800 nM protein (concentration of hexamer) was incubated with 100 μM ATP in a 10 μl reaction containing 30 mM Tris-acetate buffer (pH 6.0), 50 mM potassium acetate, 10 mM magnesium acetate, 75 mM NaCl, 1.2 mM beta-mercaptoethanol, and γ-32P ATP (0.5 μCi) as a tracer, at 55°C for 1 h, and reaction stopped by the addition of 50 mM EDTA. In reactions with DNA, 1 μM of double-stranded forked substrate DNA was used (sequence of upper strand 5′ GCTCGGTACCCGGGGATCCTCTAGAT_(20)_ -3′; sequence of lower strand 5′T_(20)_TCTAGA GGATCCCCGGGTACCGAGC-3′). In reactions carried out at different pH, Tris-acetate buffers of the same concentration but different pH were used; all other components remained the same. Reactions were analyzed by thin layer chromatography using the appropriate control reactions, as described earlier^13^. ATPase activity was calculated in terms of picomoles ATP hydrolyzed per picomole protein or picomoles ATP hydrolyzed per picomole protein per minute, as indicated on the Y axis of graphs.

### Helicase assays

Helicase assays were performed with MCM protein that had been successively subjected to TALON metal affinity-based chromatography, Strep Tactin II affinity-based chromatography and gel filtration chromatography using Superdex 200 to minimize chances of contaminating *E. coli* proteins. Activity was assayed using an M13-based forked DNA substrate that was created by annealing a radiolabelled (5′ end-labelled) 85-mer oligonucleotide (5′ TTGAACCACCC CCTTGTTAAATCACTTCTACTTGCATGCCTGCAGGTCGACTCTAGAGGATCCCCGGG TACCGAGCTCGAATTCG-3′) to single-stranded M13mp18 DNA (purchased from NEB, USA) as depicted in Fig. 3A (55 matched nucleotides and 30 nucleotide 5′ overhang). The reaction was typically carried out by incubating 500 nM MCM and 3000 nM GINS with 2.5 nM substrate DNA, in 30 mM Tris-acetate buffer (pH 4.0) containing 75 mM NaCl, 50 mM potassium acetate, 10 mM magnesium acetate, 0.1 mg/ml BSA, 5 mM ATP and 1 mM DTT. In reactions carried out at different pH, Tris-acetate buffers of the same concentration but of different pH were used, and all other components remained the same. Reactions were incubated at 55°C for 1 h, snap-chilled on ice, and stopped by the addition of bromophenol blue dye in 50 mM EDTA, 0.25% SDS, 5% glycerol. Reactions were resolved by native PAGE (6%), and the gels were subjected to autoradiography.

### Electrophoretic mobility shift assays

Electrophoretic mobility shift assays were performed as described earlier^13^. Briefly, protein was incubated with 0.1 pmol of γ-32P- radiolabelled double-stranded forked substrate DNA (same substrate as used for ATP hydrolysis assays; sequence given above), in 30 mM Tris-acetate (pH 4.0 or pH 6.0), 75 mM NaCl, 10 mM magnesium acetate, 50 mM potassium acetate, 1 mM DTT, 1 mM ATPγS and 0.1 mg/ml BSA, for 10 min. Reactions (stopped by adding bromophenol blue dye) were analyzed by native PAGE (6%) in 1X TBE buffer.

## Author Contributions

Experiments were conceived and designed by S.S., K.G. and J.A.; experiments were executed by K.G., J.A. and S.S.; data was analyzed by S.S., J.A. and K.G.; manuscript was prepared by S.S.

## Supplementary Material

Supplementary Information Supplementary Material
