# Supplementary material for: Characterization of the MCM homohexamer from the thermoacidophilic euryarchaeon Picrophilus torridus
Source: Sci Rep. 2015 Mar 12;5:9057. doi: 10.1038/srep09057 (PMC4356968; doi:10.1038/srep09057)
Supplement: Supplementary Information — Supplementary Material [file srep09057-s1.pdf]

Characterization of the MCM homohexamer from the thermoacidophilic euryarchaeon *Picrophilus torridus*

Kasturi Goswami*, Jasmine Arora* & Swati Saha #

Department of Microbiology
University of Delhi South Campus,
Benito Juarez Road,
New Delhi 110021
India.

# To whom correspondence should be addressed:
Tel: +91-9911156268
Fax: +91-11-24115270
e-mail: ss5gp@yahoo.co.in
*These two authors contributed equally to the work.

Figure S1

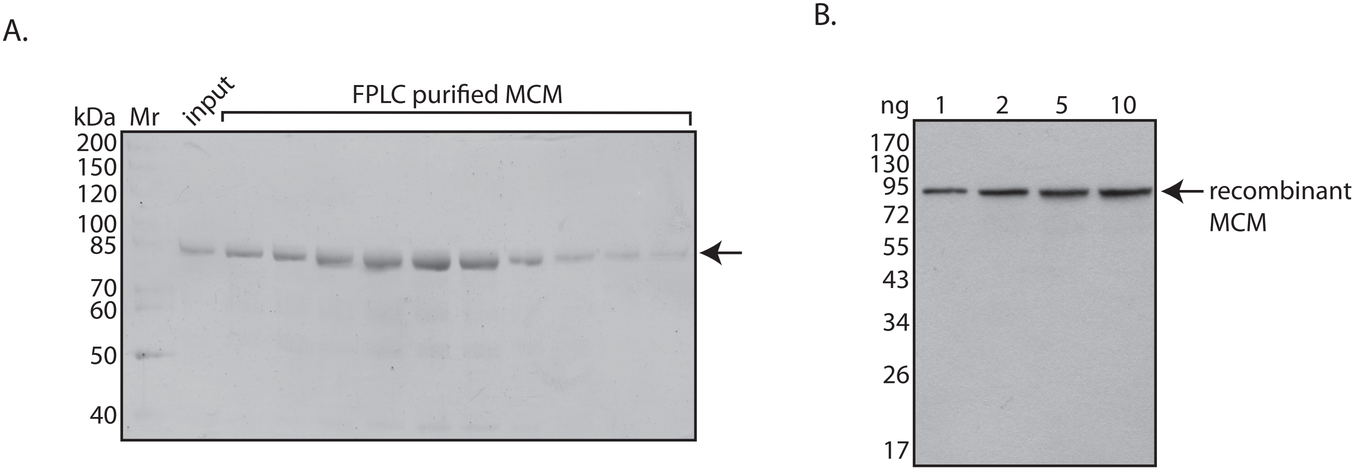

Figure S1: A. Gel filtration analysis of PtMCM. SDS-PAGE analysis of fractions matching the retention volumes shown in Figure 1C. **B.** Western blot analysis of recombinant MCM protein with mouse anti-MCM antibodies (1:1000 dilution)

Fig. S2

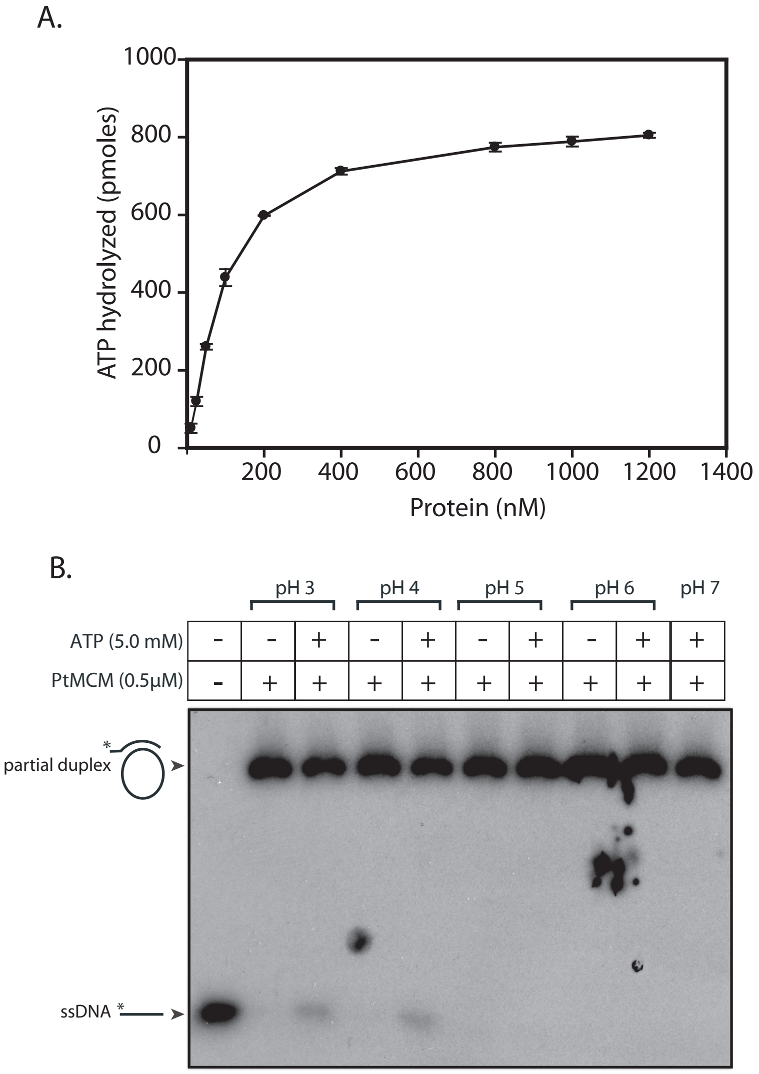

Figure S2: **A.** ATP hydrolysis as a function of protein concentration. 10-1200 nM PtMCM was incubated with 100 μM ATP in the absence of DNA at pH 6 for 1h. **B.** Helicase assay carried out in reactions of different pH using 500 nM PtMCM. Lane 1- radiolabelled ssDNA only (loaded as marker); lanes 2,4,6,8 – helicase reactions incubated in absence of ATP; lanes 3,5,7,9,10 – helicase reactions incubated in presence of ATP.

Figure S3

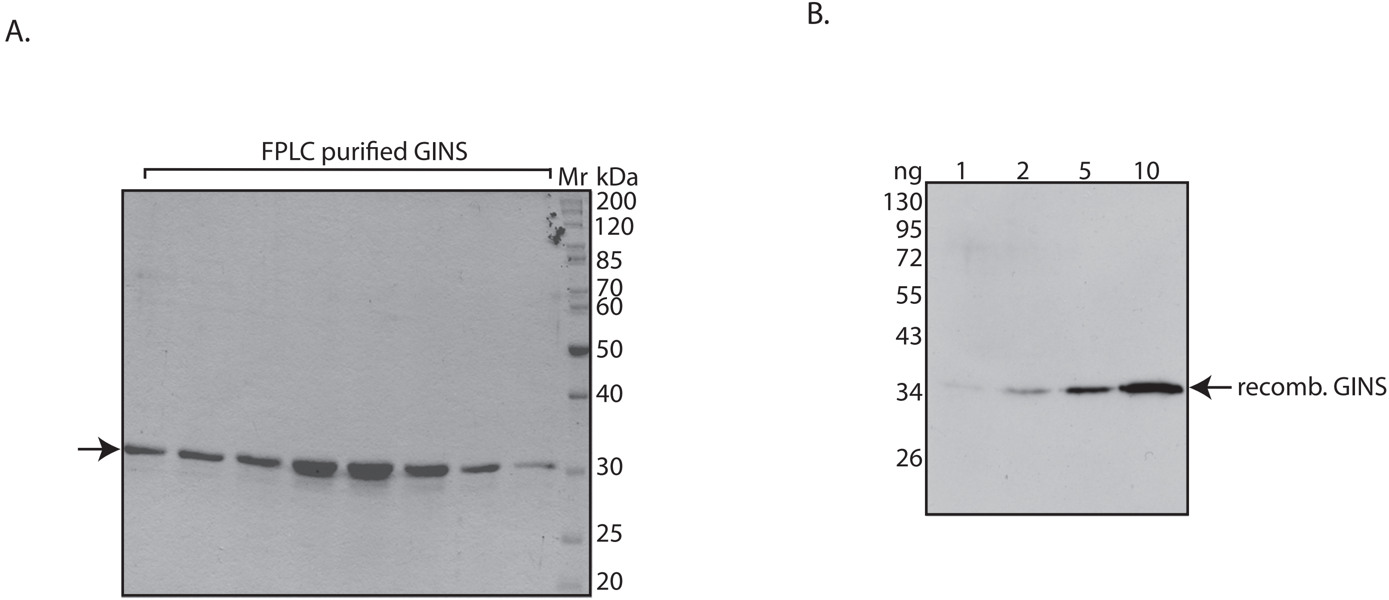

Figure S3: A. Gel filtration analysis of PtGINS. SDS-PAGE analysis of fractions matching the retention volumes shown in Figure 3C. Arrowhead indicates GINS. **B.** Western blot analysis of recombinant GINS protein with mouse anti-GINS antibodies (1:1000 dilution)

Figure S4

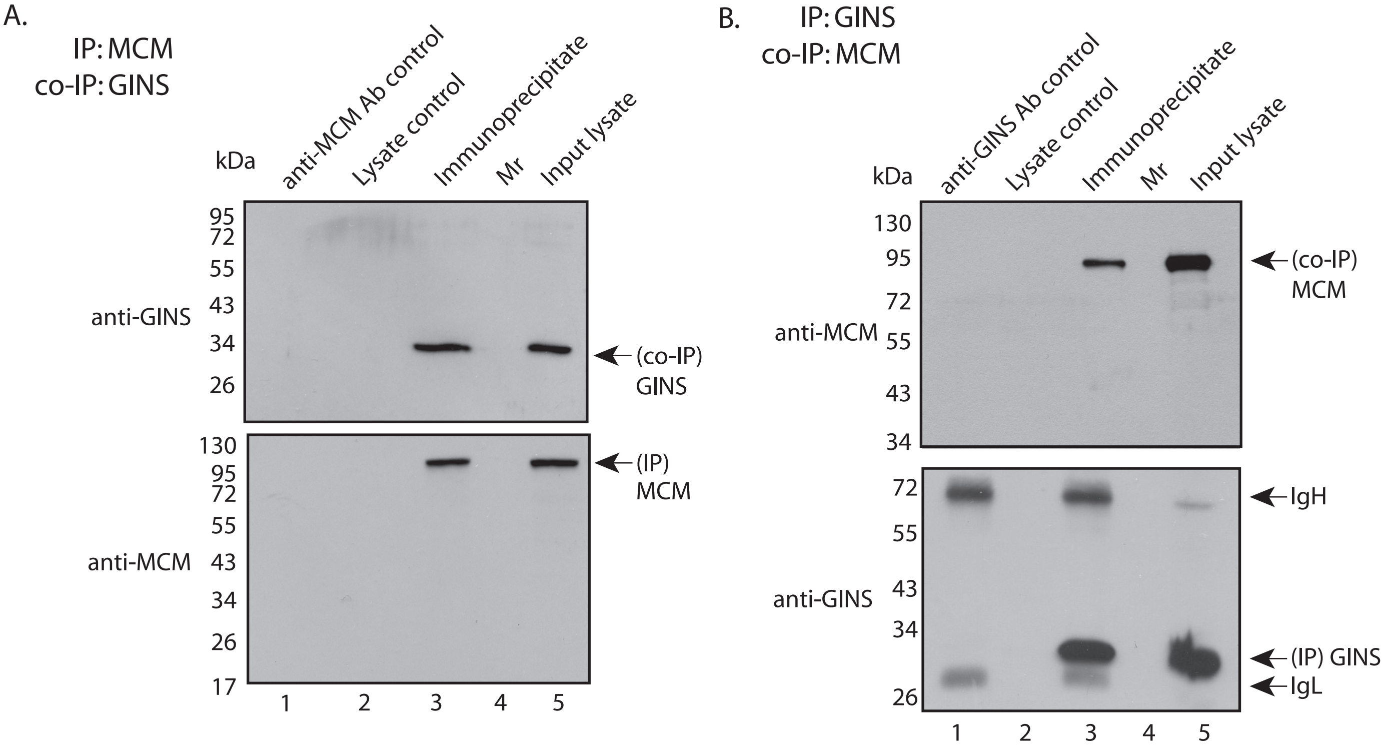

Figure S4: A & B. Immunoprecipitation analysis. **A:** PtMCM immunoprecipitates were resolved by SDS-PAGE (10%) and analyzed by western blotting using anti-MCM (to detect immunoprecipitated MCM) or anti-GINS (to detect co-immunoprecipitating GINS) antibodies (1:1000 dilution).
B: PtGINS immunoprecipitates were similarly analyzed. Lanes 1: antibody control (bead-bound antibodies only and no lysate added); lanes 2: lysate incubated with beads only (with no antibody coupled to the beads); lanes 3: immunoprecipitation reaction; lanes 4: molecular weight marker; lanes 5: input lysate.

Fig. S5

A

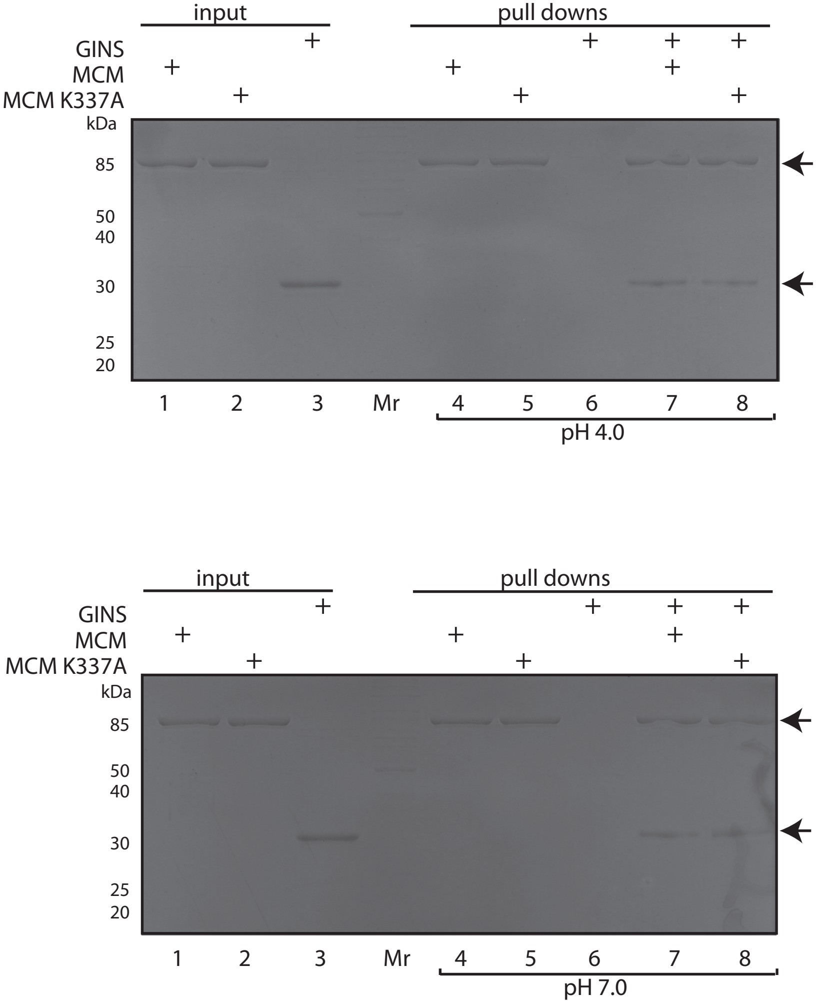

Figure S5: MCM-K337A pulldown experiment (Coomassie stain of SDS-PAGE analysis). A - at pH 4.0, B - at pH 7.0. Lanes 1-input purified PtMCM (wild type); lanes 2-input purified PtMCM-K337A; lanes 3 – input purified PtGINS; Mr - molecular weight marker; lanes 4 to 9 – analysis of eluate fractions of pulldowns performed with Strep -Tactin Superflow affinity beads. Lanes 4 - recombinant MCM alone ; lanes 5 - recombinant MCMK337A alone; lanes 6 - recombinant GINS alone; lanes 7- recombinant MCM plus recombinant GINS; lanes 8 - recombinant MCMK337A plus recombinant GINS .

Fig. S6

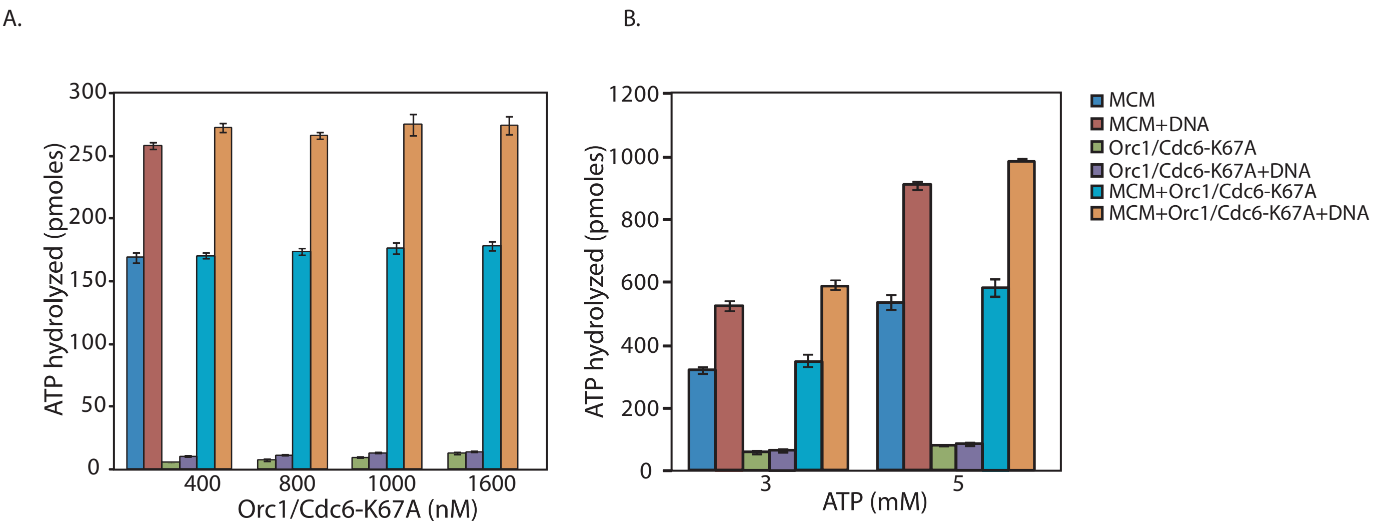

Figure S6: Effect of PtOrc1/Cdc6 on ATP hydrolysis by MCM: **A.** ATP hydrolysis by MCM as a function of PtOrc1/Cdc6-K67A. 800 nM PtMCM was incubated with 100 μM ATP in the presence or absence of DNA in Tris-acetate buffer of pH 4 for 1 h, with varying concentrations of PtOrc1/Cdc6-K67A. **B.** ATP hydrolysis by MCM (800 nM) in the presence of 3000 nM PtOrc1/Cdc6-K67A. Reactions were incubated in the presence or absence of DNA in Tris-acetate buffer of pH 4 for 1 h, either with 3 mM ATP or 5 mM ATP.
